# Analysis of patients’ request to switch from a generic drug to the original drug in external prescriptions

**DOI:** 10.1186/s40780-020-00180-w

**Published:** 2020-12-04

**Authors:** Yuhei Hamada, Masashi Uchida, Sayaka Arai, Kaori Yamazaki, Mariko Takeda, Kenichi Arai, Takako Nakamura, Takaaki Suzuki, Itsuko Ishii

**Affiliations:** 1 Division of Pharmacy, Chiba University Hospital, 1-8-1 Inohana, Chuo-ku, Chiba, 260-8677 Japan; 2 Graduate School of Pharmaceutical Sciences, Chiba University, 1-8-1 Inohana, Chuo-ku, Chiba, 260-8675 Japan

**Keywords:** Generic drug, Original drug, Inquiries, External prescription

## Abstract

**Background:**

Generic drugs are heavily promoted in Japan. The aim of this retrospective single-center study was to clarify whether the frequency and reason that patients request a switch from a generic drug to the original drug differ according to therapeutic category and dosage form.

**Methods:**

This study was performed at Chiba University Hospital. Prescription inquiries about 121 generic drugs from community pharmacies over a 3-year period (from July 2014 to June 2017) were analyzed.

**Results:**

Approximately 30% of the requests were related to the efficacy, safety, and comfort of the generic drug. The most cited motive was “patient’s desire with no reason given” at 44.5%. According to multiple logistic regression analysis, therapeutic categories and dosage forms were associated with the requests. The median request frequency differed according to therapeutic category and dosage form. The frequency was highest for “agents affecting the central nervous system” and “tablets and capsules”, respectively. Among the therapeutic categories, “agents affecting the central nervous system” had the highest median number of requests related to “decreased effectiveness”; “cardiovascular agents” had the highest median number of requests related to “physician’s instruction”; and “agents for the epidermis” had the highest median number of requests related to “uncomfortable to use”. Among dosage forms, the odds ratio for patients’ original drug request for “liniment and patch” was about 1.5 times that for “tablets and capsules”. “Liniment and patch” had the highest median frequency of requests related to “decreased effectiveness”, “uncomfortable to use”, and “patient’s desire with no reason given”.

**Conclusions:**

The request frequency and reason differed according to therapeutic category and dosage form. Pharmacists should advise each patient properly about the choice and switching of drug brands, taking into account the therapeutic category and dosage form, especially liniments and patches.

## Background

Generic drugs are bioequivalent to original drugs and less expensive, and are thus used worldwide to reduce both national medical care expenditures and patients’ medical expenses. Although some meta-analyses have provided evidence for clinical equivalence between original and generic drugs [1–4], generic drugs are still accompanied by concerns about effectiveness and safety. According to a recent systematic review, seven factors are associated with generic drug use [5]. Among them, patient-related factors consist of race, age, sex, income, insurance type or coverage, health status, and prior experiences with generic drugs [6–14]. If patients feel dissatisfied after using generic drugs, they may switch back to the original ones. The rate of switching back to the original drug is higher for patients exposed to antiepileptic drugs than for those exposed to statins, antidepressants, or β-blockers [15–17]. However, it is not clear whether the frequency and reason why patients request the original drug differ according to therapeutic category and dosage form.

In Japan, the share of generic drugs is smaller than that of other countries, at 23% in value as of 2014 and 46.9% in volume as of 2013 [18, 19]. Because Japan’s national medical expenditure is increasing year after year, the Japanese government is heavily promoting the use of generic drugs. The government set a target of a 60% share volume or higher by March 2018 [20]. To promote the use of generic drugs in Japan, generic name prescription is recommended [21]. In this system, patients can choose either the original or generic drug at community pharmacies. If the brand-name original drugs are prescribed, community pharmacists can switch to generic drugs unless the prescribing physician has specifically indicated the original drug on the prescription. If the generic name prescription system is not introduced at the hospital or clinic, generic drugs are prescribed according to the official naming convention for generic drugs marketed in Japan: the combination of the generic name of the active ingredient, the dosage form, and the name of the pharmaceutical company (e.g., carvedilol tablet 2.5 mg Pfizer). In these cases, community pharmacists should ask the prescribing physician for permission if patients desire to use the original drug for some reason. Whether or not the drugs are prescribed according to the generic name prescription system, pharmacists should advise each patient properly about drug brands to help the patients to appropriately choose their drug brand.

The aim of this study was to clarify whether the frequency and reason that patients request a switch from the generic drug to the original drug differ according to therapeutic category and dosage form using prescription inquiries from community pharmacies.

## Methods

### Observation period and target drugs

This retrospective single-center study was performed at Chiba University Hospital, near Tokyo. This hospital has 850 beds and 35 clinical departments and provides medical care for seriously ill patients referred from other medical institutions. The number of outpatients per day was about 2200 in 2017.

The observation period was from July 2014 to June 2017. Original drugs which were prescribed more in volume in a year were preferentially switched to generic drugs. During the observation period, 170 original drugs for outpatients were switched to generic drugs. For cases in which two or more original drugs using a single active ingredient in the same dosage form had previously been adopted, all brand-name original drugs were switched to a single generic drug (e.g., Amlodin® OD 5 mg tablet and Norvasc® 5 mg tablet were switched to a generic amlodipine besylate 5 mg tablet). Therefore, 170 original drugs were switched to 167 generic drugs. To analyze prescription inquiries about generic drugs, generic drugs that contained different amounts of an active ingredient were aggregated as the same drug in this study (e.g., carvedilol 1.25 mg, 2.5 mg, and 10 mg tablets). Consequently, the number of target generic drugs examined was 121.

The therapeutic categories of each drug were determined based on the Japanese standard commodity classification [22]. Drugs belonging to two or more categories were classified into the most-often prescribed category. As for dosage forms, target generic drugs were categorized into internal, external, and injection drugs. Next, each group was further subclassified. Internal drugs were subclassified into “tablets and capsules”, powder form drugs (“powder, granules, and dry syrup”), and “liquid medicine” with significantly different properties. External drugs were subclassified into “liniments and patches” and “other external drugs” (eye drops, inhaler, suppository, enema, and gargling). There were no injection drugs in this study.

### Method of recording prescription inquiries from community pharmacies

The generic name prescription system has not been introduced at Chiba University Hospital. Physicians prescribed generic drugs adopted at Chiba University Hospital for outpatients as well as for inpatients. Physicians prescribed drugs by using an ordering system connected to electronic medical records, and prescriptions for nearly all outpatients were filled by community pharmacies. If patients desired to use the original drug for some reason, community pharmacists should ask the prescribing physician for permission. In these cases, community pharmacists telephoned the hospital pharmacy. When hospital pharmacists received prescription inquiries regarding a desire to switch from a generic drug to the original drug, they asked the community pharmacists for the reason. Then, hospital pharmacists asked the prescribing physicians about these inquiries and relayed the answers to the pharmacies. The details of the inquiries and the physician’s answers were recorded in the electronic medical record system and in an “inquiry database” created in Microsoft® Excel. This database was used for the following analyses.

### Classification of prescription inquiries about generic drugs from community pharmacies

Prescription inquiries about generic drugs from community pharmacies were categorized as follows: “the pharmacy wants to switch to the original drug because there is no stock of the generic drug”, “patient’s desire to switch to the original drug”, “request to switch to another generic brand”, “prescription error due to the switch from the original to a generic drug”, and “others”. Next, the category “patient’s desire to switch to the original drug” was further subclassified as follows: (1) decreased effectiveness; (2) adverse event; (3) uncomfortable to use (e.g., the generic patch was more difficult to remove from the affected area than the original drug); (4) the patient felt the generic drug was not suitable for them; (5) negative experience with the use of another (generic) drug in the past; (6) medication-related problems (e.g., avoidance of incorrect administration due to a change in appearance or name); (7) patient’s desire with no reason given; and (8) physician’s instruction.

### Calculation of the frequency of requesting a switch to the original drug for each generic drug

Among inquiries on each generic drug categorized as “patient’s desire to switch to the original drug”, if there were two or more inquiries from the same patient for the same reason, it was regarded as one inquiry and duplicates were excluded (variable A). The number of patients prescribed each generic drug during the observation period was extracted from the electronic medical record system and repeat prescriptions for the same patients were excluded for each drug (variable B). The frequency of requesting a switch to the original drug, assuming that each generic drug was prescribed to 1000 patients, was calculated by the following equation: (A/B) × 1000.

### Statistical analysis

Correlations were measured using Spearman’s correlation coefficient test. Multiple comparison testing was performed with the Steel-Dwass test. Multiple logistic regression analysis was performed to test the relationship between therapeutic categories or dosage forms and the presence of inquiries designated “patient’s desire to switch to the original drug” using SPSS version 24 (IBM Corp., Armonk, NY). Statistical significance was defined as *P* < 0.05.

## Results

### Outline of prescription inquiries about generic drugs from community pharmacies

In total, the 124 original drugs listed in Table S1 were sporadically switched to 121 generic drugs during the observation period. There were 3378 inquiries about generic drugs from community pharmacies, which were classified as follows: 1638 cases (48.5%) for “the pharmacy wants to switch to the original drug because there is no stock of the generic drug”; 1541 cases (45.6%) for “patient’s desire to switch to the original drug”; 105 cases (3.1%) for “request to switch to another generic brand”; 35 cases (1.0%) for “prescription error due to the switch from the original to a generic drug”; and 59 cases (1.7%) for “others”.

### Reasons that patients requested to switch from generic drugs to original drugs

**Fig. 1:**
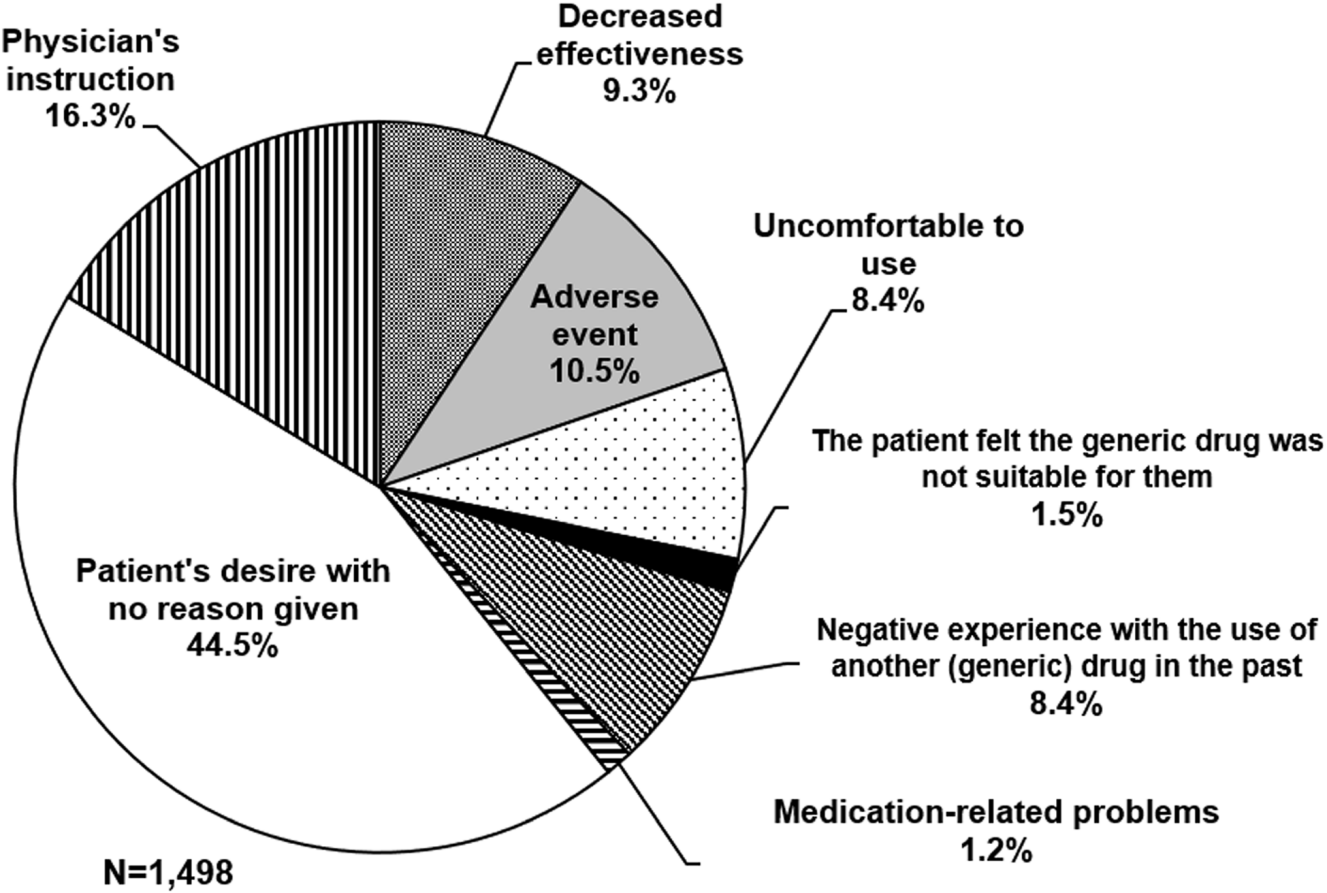
Reasons for requesting to switch from generic drugs to the original drugs

More specific reasons were classified in the category “patient’s desire to switch to the original drug”. The number of patients prescribed the target generic drugs, excluding duplications, was 109,296, and the number of patients who desired to switch to the original drug, excluding duplications, was 1498 (1.4%). The percentages of each reason that patients gave for requesting to switch to the original drug are shown in Fig. 1. The most common reason was “patient’s desire with no reason given” at 44.5%. A total of 444 requests (29.6%) were related to the efficacy, safety, and comfort of the generic drugs, comprising “decreased effectiveness”, “adverse event”, “uncomfortable to use”, and “the patient felt the generic drug was not suitable for them”. Although some cases of decreased effectiveness and adverse events caused by generic drugs were assessed by physicians, most cases were based on patient’s claims and their veracity was unknown. In addition, 126 requests (8.4%) were considered to be due to a past negative experience with the use of another drug, either generic or original. The percentage of requests in which the physician hoped to prescribe the original drug was 16.3%.

### Correlation among days after introducing each generic drug, number of patients prescribed each generic drug, and number of patients requesting original drug

One generic drug was not prescribed during the observation period and no request to switch to the original drug was received for 23 generic drugs. Statistically significant positive correlations were observed between the number of days after the introduction of each generic drug and the number of patients prescribed each generic drug (Fig. S1A). There was also a strong positive correlation between the number of patients prescribed each generic drug and the actual number of patients who desired to switch to the original drug for each generic drug (Fig. S1B). There was no correlation between the number of patients prescribed each generic drug and the frequency of the switch request per 1000 patients (Fig. S1C). Notably, there was no correlation between the number of days after the introduction of each generic drug and the frequency of the switch request per 1000 patients (Fig. S1D). Therefore, it was considered appropriate to analyze all the target generic drugs together, even though the number of patients who desired to switch to the original drug may change in a time-dependent manner after the introduction of each generic drug.

### Frequency and reason for requesting to switch to the original drug according to therapeutic category and dosage form

**Fig. 2:**
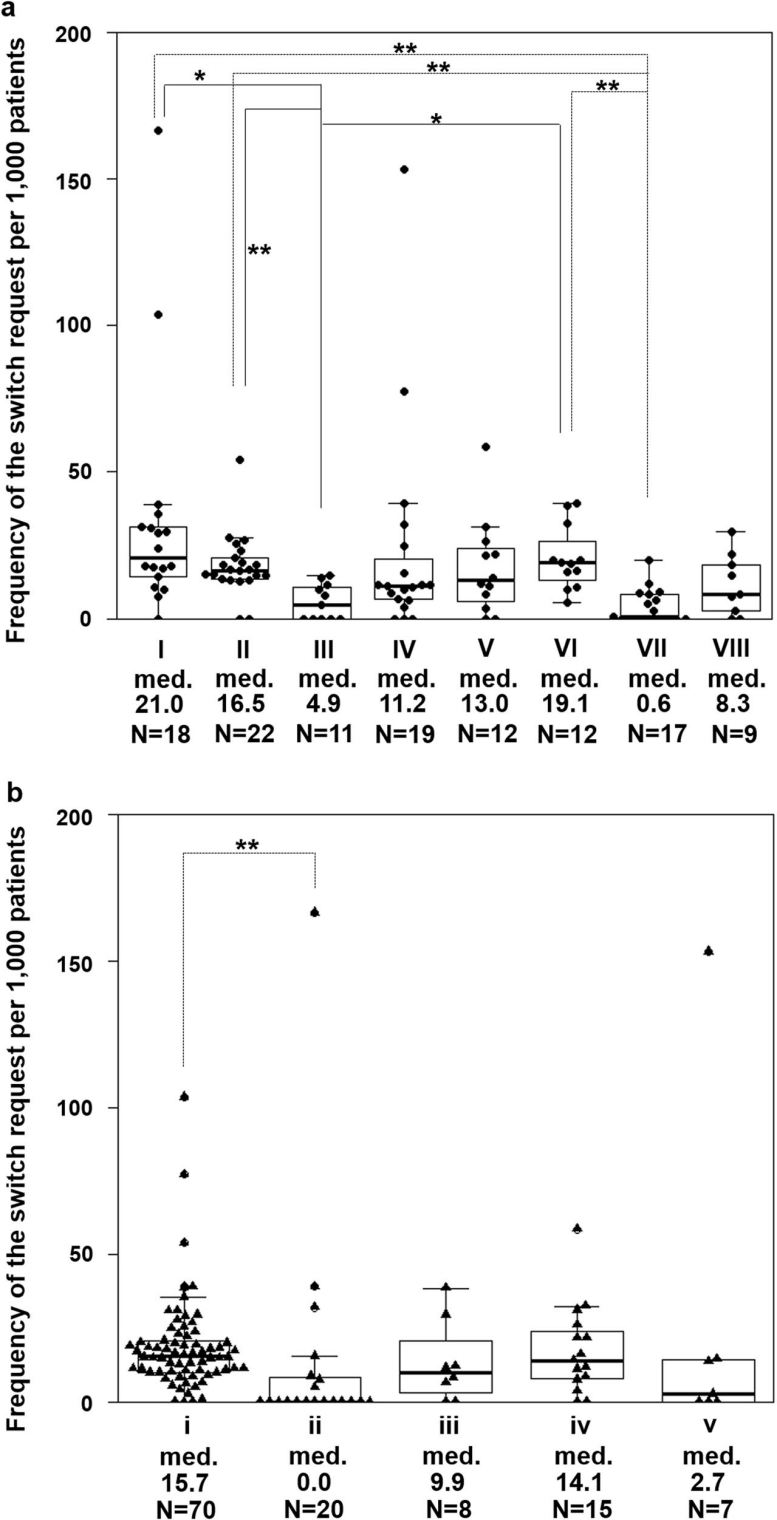
Frequency of the switch request per 1000 patients for each generic drug. **a**. Therapeutic category. I, agents affecting the central nervous system; II, cardiovascular agents; III, agents affecting respiratory organs; IV, agents affecting digestive organs; V, agents for the epidermis; VI, agents affecting metabolism; VII, agents against pathogenic organisms and parasites; VIII, other agents. **b**. Dosage form. i, tablets and capsules; ii, powder, granules, and dry syrup; iii, liquid medicine; iv, liniments and patches; v, other external drugs (eye drops, inhaler, suppository, enema, and gargling).The line in the box represents the median. The upper and lower ends of the box represent the third and first quartiles, respectively. The upper and lower ends of the whiskers represent the maximum and minimum values excluding outliers. Med., median. *P < 0.05, **P < 0.01

The median frequency of requests per 1000 patients was 13 (range, 0–167). All data are described in Table S2. Figure 2 shows the request frequency according to therapeutic category and dosage form. The median frequencies, from high to low, were as follows: “agents affecting the central nervous system”, “agents affecting metabolism”, “cardiovascular agents”, and “agents for the epidermis” (Fig. 2a). For dosage forms, the median was highest for “tablets and capsules” (Fig. 2b). According to multiple logistic regression analysis, therapeutic categories and dosage forms were associated with the presence of the inquiry “patient’s desire to switch to the original drug” (Table 1). There were significant differences among therapeutic categories and dosage forms. Among therapeutic categories, “agents affecting respiratory organs” and “agents against pathogenic organisms and parasites” were less likely to be requested than “agents affecting the central nervous system”. Among dosage forms, the odds ratio for patients requesting to switch to the original drugs for “liniments and patches” was about 1.5 times that for “tablets and capsules”.

**Table 1:** Results of multivariate analysis

	n	OR	95% CI	*P*
**Therapeutic category**				
Agents affecting the central nervous system^a^	18,845	1.00		
Cardiovascular agents	14,036	1.06	0.89–1.26	0.495
Agents affecting respiratory organs	8237	0.57	0.44–0.74	< 0.001*
Agents affecting digestive organs	25,773	0.93	0.80–1.09	0.376
Agents for the epidermis	11,929	0.87	0.62–1.23	0.432
Agents affecting metabolism	9767	0.85	0.67–1.08	0.185
Agents against pathogenic organisms and parasites	12,373	0.29	0.22–0.39	< 0.001*
Other agents	8336	0.63	0.47–0.84	0.002*
**Dosage form**				
Tablets and capsules^a^	82,217	1.00		
Powder, granules, and dry syrup	2321	0.81	0.51–1.26	0.347
Liquid medicine	3017	0.80	0.57–1.14	0.213
Liniments and patches	16,110	1.49	1.11–2.00	0.009*
Other external drugs	5631	0.82	0.59–1.15	0.260

*CI* confidence interval; *OR* odds ratio

^a^Reference category

* Indicates significance at *P* = 0.05

Table 2 shows the reasons for requesting to switch to the original drug according to therapeutic category and dosage form. “Patient’s desire with no reason given” was the most frequent request in all therapeutic categories and dosage forms. Among the therapeutic categories, “agents affecting the central nervous system” had the highest median frequency related to “decreased effectiveness”; “cardiovascular agents” had the highest median frequency related to “physician’s instruction”; and “agents for the epidermis” had the highest median frequency related to “uncomfortable to use”. Among the dosage forms, “liniments and patches” had the highest median frequency related to “decreased effectiveness”, “uncomfortable to use”, and “patient’s desire with no reason given”.

**Table 2:**
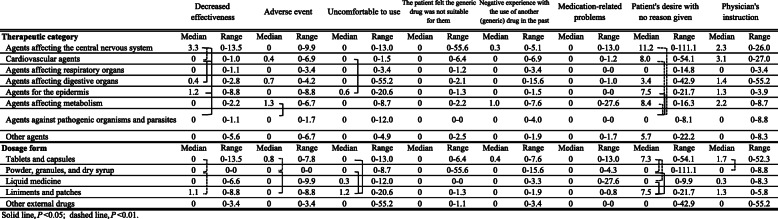
Reasons for requesting to switch to the original drug for each generic drug

## Discussion

This study demonstrated that the median request frequency and reason differed according to therapeutic category and dosage form. The main strength of the study is that the exact numbers of patients prescribed each generic drug, excluding duplicates, were used for the analysis. This allowed the frequency of requests to be calculated for each generic drug. In contrast, it should be noted that the frequency of requests could vary due to a few requests for less frequently prescribed drugs, and this could affect the results of Fig. 2 and Table 2.

In this study, the percentage of patients who desired to switch from a generic drug to the original drug was 1.4% of all patients prescribed generic drugs, excluding duplicates. This percentage was lower than that in a survey conducted from 2014 to 2016 in Japan in which the percentage of patients who answered “never want to use generic drugs even if individual payments gets cheaper” ranged from 11.9 to 13.7% [21]. Although it has been reported that there is no statistically significant difference in the willingness to use generic drugs by region in Japan [7], the cause of this discrepancy was unknown. Recommendations by physicians and pharmacists affect patients’ use of generic drugs [6, 23]. In this study, patients’ negative attitude toward generic drugs might be improved through a proper explanation of generic drugs by the medical professionals involved.

A previous study conducted in a different region in Japan from that in the present study investigated the prescriptions received by 233 community pharmacies in which generic drugs were not allowed by physicians [24]. The proportion of the therapeutic categories of the refused drugs was in the following order (from high to low): cardiovascular agents, agents affecting the central nervous system, agents affecting digestive organs, and agents for the epidermis. As shown in Fig. S1B, the switch requests tended to increase as the number of patients prescribed the drug increased. Even though the results of the previous study were not adjusted for the number of patients, they were similar to those of our study (Fig. 2a). Therefore, the same findings were obtained in other settings in Japan, although the present work was a single-center study.

It was previously reported that the rate of switching back to the original drug was higher for antiepileptic drugs than for other therapeutic categories [15–17]. In the present study, which did not include antiepileptic drugs, “agents affecting the central nervous system” had the highest median frequency of requests among the therapeutic categories (Fig. 2a). These agents also had the highest median frequency related to “decreased effectiveness” (Table 2). This may be because patients could subjectively assess the effects of analgesics, sleeping pills, and neuropsychiatric drugs.

Among dosage forms, the odds ratio for patients requesting the original drugs for “liniments and patches” was about 1.5 times that for “tablets and capsules” (Table 1). “Liniments and patches” and/or “agents for the epidermis” had the highest median frequency of requests related to “decreased effectiveness” and “uncomfortable to use” (Table 2). This was probably because it was easy for patients to judge the local efficacy, such as the analgesic effect, of liniments and patches. Differences have been reported in the physical and pharmaceutical properties of tape preparations among brand-name original drugs and generic drugs, and these differences could influence patients’ comfort [25, 26]. In the present study, these differences in properties among drug brands would have led to the patients’ requests to switch. More careful explanations should be required when pharmacists provide liniments and patches as generic drugs to patients.

One of the seven factors associated with generic drug use is physician-related factors [5]. Physician specialty, physician age, and resident’s experience level influence generic drug use [27–29]. However, it is not clear whether the use of generic drugs by physicians differs according to therapeutic category and dosage form. In a survey conducted in France, physicians often used “not for generic substitution” when prescribing thyroid hormones (“other agents” in the present study), antiepileptic drugs (“agents affecting the central nervous system”), and antiplatelet agents (“agents affecting metabolism”) [30]. The main or frequent reason given for not substituting thyroid hormones and antiepileptic drugs was biological or clinical inequivalence to original drug [30]. As shown in Table 2, the median request frequency due to “physician’s instruction” was relatively high for “cardiovascular agents”, “agents affecting the central nervous system”, and “agents affecting metabolism”. There were several drugs in these categories in which management of blood concentration was recommended, suggesting that physicians might have avoided the risk of an alteration in efficacy and safety associated with a switch to generic drugs.

In more than 40% of inquiries, the reason(s) that patients wanted to use the original drug were unclear in this study (Fig. 1). These undefined results seemed to be due to the following three reasons: (1) the patients had no clear reason to avoid the generic drug, (2) the patients actually had clear reasons but did not tell the community pharmacists, and (3) the community pharmacists did not sufficiently interview the patients. In Japan, reasons such as “concerns about efficacies and adverse effects”, “negative information about qualities and efficacies of generic drugs from news media”, and “discouraged by family or friends” were reported as reasons for patients not wanting to use generic drugs [21]. Some studies have shown that patients obtain information on generic drugs in Japan and in other countries from not only their physicians and pharmacists, but also the media and Internet [23, 31, 32]. Patients can easily get information on generic drugs on the Internet, but some websites contain incomplete or inaccurate information [33]. It was presumed that patients who wanted to switch to the original drug in this study might have been influenced by information from the media, Internet, or people with whom they associate.

Among the seven factors associated with generic drug use, patient-related factors consist of race, age, sex, income, insurance type or coverage, health status, and prior experiences with generic drugs [5]. There are few reports about Japanese. Kobayashi et al. reported in 2011 that age and sex are not related to the willingness for generic drug substitution [7]. However, in a survey conducted in 2016, the percentage of patients who answered “do not want to use generic drugs if possible” ranged from 5.7 to 7.1% under the age of 39 years old, whereas the percentage ranged from 11.3 to 15.4% over the age of 40 [21]. It cannot be denied that the willingness for generic drug use differs depending on age in Japan. This survey also shows that the willingness for generic drug use differs depending on out-of-pocket medical fee [21]. Moreover, Kobayashi et al. reported that patients’ past experiences of generic drug use were associated with the willingness for generic drug substitution [7]. These factors were not analyzed in this study and may associate with the request to switch from a generic drug to the original drug in the present study.

This study was performed using data from 2014 to 2017. The circumstances surrounding generic drugs have changed in the last few years in Japan. The generic drug share volume increased dramatically from 46.9% in 2013 to 72.6% in 2018 in Japan [18]. The generic drug share volume at Chiba University Hospital was 86.8% as of 2017. As mentioned above, patients’ past experiences of generic drug use were associated with the willingness for generic drug substitution in Japan (Odds ratio: 2.93) [7]. The percentage of patients who had an experience with generic drugs in Japan increased from 76.6% in 2013 to 86.5% in 2016 [21], and will be higher in 2020. On the other hand, the willingness for using generic drug has not changed significantly between 2013 and 2016; the highest at 77.7% in 2014 and the lowest at 71.0% in 2016 [21], although there is no data since 2017. Consequently, we expected that the results of the present study may reflect the current situation although this study was performed using data from 2014 to 2017.

This study has the following limitations. First, if a physician wrote the prescription for an original drug by hand, the community pharmacist might not have inquired about the drug requested. Second, because the brands of generics might have been different at community pharmacies, it was unclear whether the problems with generic drugs were associated with the particular brands adopted by our hospital. Third, we could not determine whether the patients had continually used the original drugs before the adoption of generic drugs. In a survey conducted in 2016, there were some patients who did not want to use generic drugs because they hope to use drugs familiar with taking [21]. This suggests that the present study may include some patients who wanted to avoid changing familiar drugs rather than avoiding generic drugs. Finally, the reason for the request was provided by community pharmacists on behalf of patients themselves.

## Conclusions

This study demonstrated that the frequency and reason for requesting a switch to an original drug differed according to therapeutic category and dosage form. Pharmacists should advise each patient properly on their options regarding drug brands, taking into account therapeutic category and dosage form, especially in regard to liniments and patches. Extensive research is needed to further generalize the findings of this study.

## Supplementary Information

**Additional file 1: Table S1.** Original drugs switched to generic drugs

**Additional file 2: Figure S1.** Correlation among days after introducing each generic drug, number of patients prescribed each generic drug, and number of patients requesting original drug. A. Correlation between the number of days after the introduction of each generic drug and the number of patients prescribed each generic drug. B. Correlation between the number of patients prescribed each generic drug and the actual number of patients who desired to switch to the original drug. C. Correlation between the number of patients prescribed each generic drug and the frequency of the switch request per 1000 patients. D. Correlation between the number of days after the introduction of each generic drug and the frequency of the switch request per 1000 patients.

**Additional file 3: Table S2.** Data set of the manuscript

## Data Availability

All data generated or analyzed in this study are included within the article.
