# Supplementary material for: Analysis of patients’ request to switch from a generic drug to the original drug in external prescriptions
Source: J Pharm Health Care Sci. 2020 Dec 4;6:27. doi: 10.1186/s40780-020-00180-w (PMC7716439; doi:10.1186/s40780-020-00180-w)
Supplement: Supplementary file 1 — Additional file 1: Table S1. Original drugs switched to generic drugs [file 40780_2020_180_MOESM1_ESM.docx]

**Table S1.** **Original drugs switched to generic drugs**
