# Supplementary material for: Analysis of patients’ request to switch from a generic drug to the original drug in external prescriptions
Source: J Pharm Health Care Sci. 2020 Dec 4;6:27. doi: 10.1186/s40780-020-00180-w (PMC7716439; doi:10.1186/s40780-020-00180-w)
Supplement: Supplementary file 2 — Additional file 2: Figure S1. Correlation among days after introducing each generic drug, number of patients prescribed each generic drug, and number of patients requesting original drug. A. Correlation between the number of days after the introduction of each generic drug and the number of patients prescribed each generic drug. B. Correlation between the number of patients prescribed each generic drug and the actual number of patients who desired to switch to the original drug. C. Correlation between the number of patients prescribed each generic drug and the frequency of the switch request per 1000 patients. D. Correlation between the number of days after the introduction of each generic drug and the frequency of the switch request per 1000 patients. [file 40780_2020_180_MOESM2_ESM.docx]

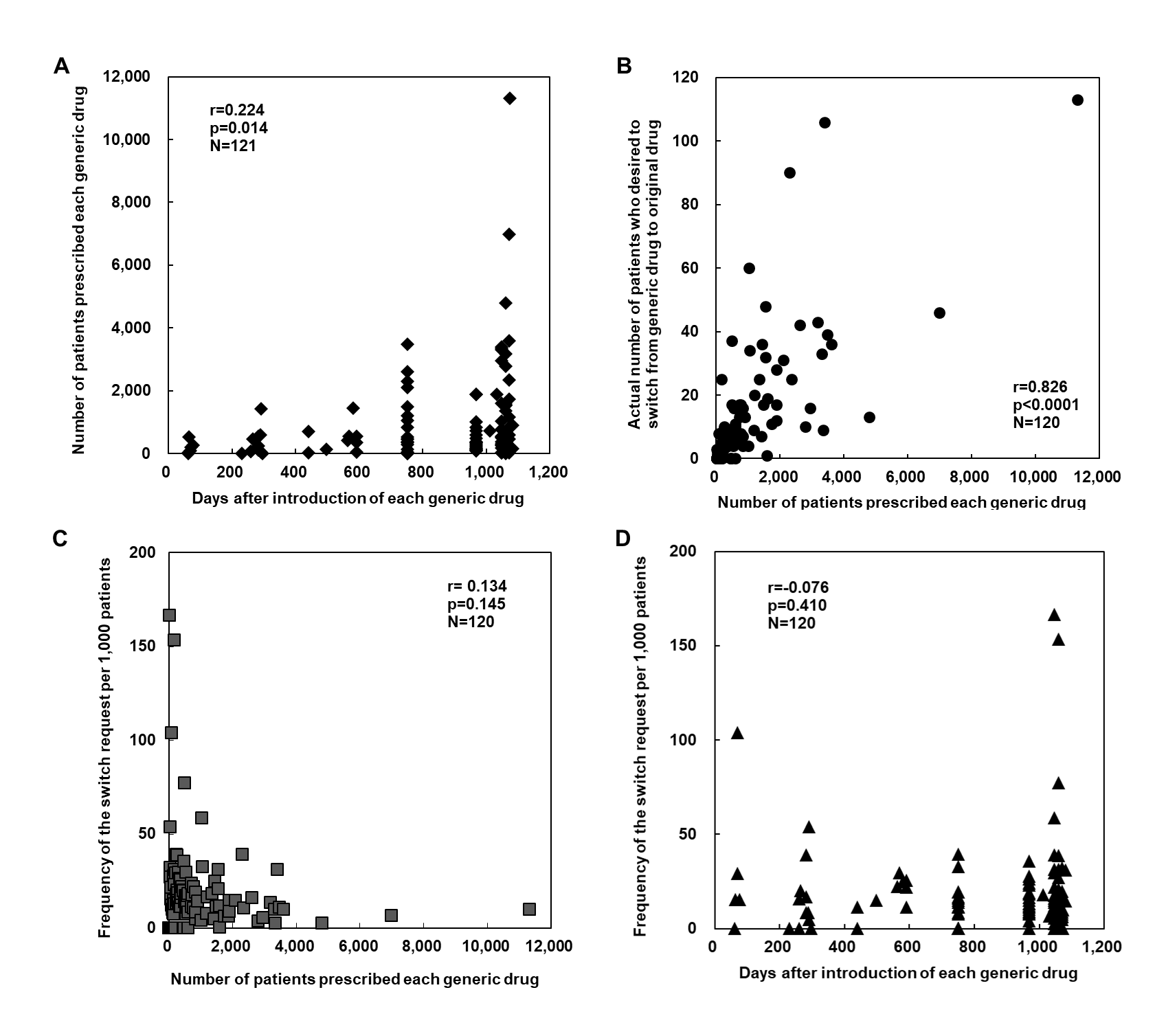


**Fig. S1 Correlation among days after introducing each generic drug, number of patients prescribed each generic drug, and number of patients requesting original drug.**

A. Correlation between the number of days after the introduction of each generic drug and the number of patients prescribed each generic drug. B. Correlation between the number of patients prescribed each generic drug and the actual number of patients who desired to switch to the original drug. C. Correlation between the number of patients prescribed each generic drug and the frequency of the switch request per 1,000 patients. D. Correlation between the number of days after the introduction of each generic drug and the frequency of the switch request per 1,000 patients.
